# Improving CPAP Adherence in Adults With Obstructive Sleep Apnea Syndrome: A Scoping Review of Motivational Interventions

**DOI:** 10.3389/fpsyg.2021.705364

**Published:** 2021-08-12

**Authors:** Giada Rapelli, Giada Pietrabissa, Gian Mauro Manzoni, Ilaria Bastoni, Federica Scarpina, Ilaria Tovaglieri, Elisa Perger, Sergio Garbarino, Paolo Fanari, Carolina Lombardi, Gianluca Castelnuovo

**Affiliations:** ^1^Department of Psychology, Catholic University of the Sacred Heart, Milan, Italy; ^2^Psychology Research Laboratory, Istituto Auxologico Italiano IRCCS, Milan, Italy; ^3^Faculty of Psychology, eCampus University, Novedrate, Italy; ^4^U. O. di Neurologia e Neuroriabilitazione, Istituto Auxologico Italiano IRCCS, Verbania, Italy; ^5^“Rita Levi Montalcini” Department of Neuroscience, University of Turin, Turin, Italy; ^6^Pulmonary Rehabilitation Department, Istituto Auxologico Italiano IRCCS, Verbania, Italy; ^7^Department of Cardiovascular, Neural and Metabolic Sciences, Sleep Disorders Center, Instituto Auxologico Italiano IRCCS, Milan, Italy; ^8^Department of Neuroscience, Rehabilitation, Ophthalmology, Genetics and Maternal-Infantile Sciences, University of Genoa, Genoa, Italy; ^9^Department of Medicine and Surgery, University of Milano-Bicocca, Milan, Italy

**Keywords:** sleep disorders, obstructive sleep apnea syndrome, continuous positive airway pressure, adherence, motivational intervention

## Abstract

**Objective:** This scoping review aims to provide an accessible summary of available evidence on the efficacy of motivational interventions to increase adherence to Continuous Positive Airway Pressure (CPAP) among patients with Obstructive Sleep Apnea Syndrome (OSAS) and of their specific aspects and strategies by assessing adherence measures.

**Methods:** A literature search was performed in PubMed, Scopus, Medline, PsycINFO, and Web of Science databases using the concepts of “obstructive sleep apnea syndrome,” “continuous positive airway pressure,” “motivational intervention,” and “adherence.” Rigorous inclusion criteria and screening by at least two reviewers were applied. Data were extracted to address the review aims and were presented as a narrative synthesis.

**Results:** Search for databases produced 11 randomized controlled trials, all including naïve CPAP users. Findings showed that motivational interventions were more effective than usual care and educational programs in increasing adherence to CPAP, despite results were not always maintained over time across studies.

**Discussion:** To our knowledge, this is the first scoping review of the literature aimed to explore the characteristics and impact of motivational interventions to promote adherence to CPAP in patients with OSAS. More research providing a detailed description of motivational strategies, and testing of their association with positive treatment outcomes via both direct and indirect measures are needed to increase awareness on active mechanisms of change.

## Introduction

Obstructive Sleep Apnea Syndrome (OSAS) is a sleep-related breathing disorder characterized by transient interruption of ventilation during sleep caused by complete or partial occlusion of the upper airway (McNicholas et al., 2007). Consequent oxygen desaturation, increased inspiratory effort, sleep fragmentation, and arousal from sleep (Lévy et al., 2015; Jennum et al., 2021) lead to excessive daytime sleepiness, cardiovascular, and metabolic diseases (Pépin et al., 2019; Visniauskas et al., 2021), cognitive and memory impairments (Alomri et al., 2021; Huang et al., 2021; Legault et al., 2021), and mood disorders (Garbarino et al., 2020).

Continuous Positive Airway Pressure (CPAP) is the treatment of choice for moderate to severe OSAS (Zhang et al., 2015; Chen et al., 2021; Sugiyama et al., 2021). It involves the use of an airflow generator which provides a constant stream of pressurized air to splint open and maintain patency of the upper airways during the inspiratory and expiratory phases of breathing. However, its effectiveness is limited by poor acceptance and adherence (Bros et al., 2020). The literature suggests that 8 to 15% of patients with OSAS refuse CPAP treatment after the first night and that at least 50% of individuals discontinue its usage within 1 year from the treatment beginning (Rotenberg et al., 2016; Borker et al., 2021; Contal et al., 2021).

A dose-response relationship between CPAP adherence and improvements in health and quality of life has been highlighted in several studies, which show a substantial increase in memory, functional status, and blood pressure (Gay et al., 2006; Giles et al., 2006), as well as reduced rates of sleepiness and cardiovascular mortality (Marin et al., 2005; Martínez-García et al., 2012; Dong et al., 2013; Mashaqi and Gozal, 2020) among those who use the device for a greater number of hours per night. However, what remains unclear is the nightly duration of CPAP usage required to normalize functioning (Lewis et al., 2004), which ranges from a minimum of 4 h (Lewis et al., 2004; Richard et al., 2007) to 6–8 h per night (Zimmerman et al., 2006; Weaver et al., 2007) across studies.

Specific barriers to treatment include skin irritation, dry throat, nasal congestion, and mask leaks (Zozula and Rosen, 2001; Cayanan et al., 2019), but a reduction in side effects offered by technical solutions (i.e., air humidifiers and different types of devices) did not correlate with increased adherence to CPAP, and quality of life in several investigations (Zozula and Rosen, 2001; Weaver and Grunstein, 2008; Broström et al., 2009; Sawyer et al., 2011). Adherence to CPAP use might, therefore, depends on factors other than disease-specific characteristics or technological advancements in the delivery of positive airway pressure, including psychological, motivational, and environmental aspects. These encompass characteristics of negative affectivity and social inhibition associated with Type-D (distressed) personality (Broström et al., 2007; Copur et al., 2018), or symptoms of claustrophobia (Chasens et al., 2005), while research findings are inconclusive on the association between mood disorders, such as anxiety and depression, and adherence to CPAP (Stepnowsky et al., 2002; Garbarino et al., 2018; Yang et al., 2020; Scarpina et al., 2021). Additional factors include coping styles with challenging situations (active vs. passive), treatment expectations, and perceived self-efficacy (i.e., confidence in one's own ability to carry out a particular behavior) (Aloia et al., 2005; Olsen et al., 2008; Baron et al., 2011; Sawyer et al., 2011; Mehrtash et al., 2019), together with social support (Lewis et al., 2004; Xu et al., 2020), and marital satisfaction (i.e., problems with the bed partner) (Batool-Anwar et al., 2017; Luyster, 2017; Gentina et al., 2019).

Accordingly, findings from previous studies highlight that interventions focused on enhancing knowledge about OSAS and CPAP use, or on removing a potential barrier to device usage lead only to partial improvements in treatment adherence (Aloia et al., 2004; Minassian and Doran, 2020).

Indeed, behavioral change is a complex process involving three specific constructs: (a) readiness to change, (b) perceived importance of change, and (c) confidence in one's ability to change (Miller and Rollnick, 2002, 2013).

Several theoretically informed behavior change interventions targeting the individuals' motivation to change (Miller and Rollnick, 1991, 2002; Kreman et al., 2006; Pietrabissa et al., 2015) and self-efficacy (Miller et al., 1997; Pietrabissa et al., 2013) to improve adherence to treatment recommendations have been developed and successfully tested across chronic conditions (Burke et al., 2003; Pietrabissa et al., 2012, 2017; Soderlund, 2018), including pulmonary disease that requires the use of the CPAP (Aloia et al., 2005; Weaver and Grunstein, 2008; Shannon et al., 2017).

However, motivational interventions vary widely across studies due to different theoretical backgrounds and employed strategies, Moreover, adherence is operationalized and measured differently across studies—thus limiting the conceptualization of behavioral change interventions in clinical practice and the generalization of research findings (Martin et al., 2005).

To overcome this gap, this study was conducted to systematically review the research done in this area, as well as to provide an accessible summary of available evidence on motivational interventions to increase adherence to CPAP use among patients with OSAS by answering the following research questions: (1) Which are the characteristics of motivational interventions to increase CPAP use/acceptance/adherence in patients with OSAS? (2) Which motivational strategies are specifically used to enhance adherence to CPAP in patients with OSAS? (3) Which theoretical model underlines the interventions? (4) Who provides the intervention? (5) How adherence to CPAP is operationalized and measured, and what are the reported effects (primary and secondary outcomes) of motivational interventions in the short- and long-term?

## Methods

This scoping review employed the five-stage framework as outlined by Arksey and O'Malley (2005) as follow: (1) identifying the research question, (2) identifying relevant studies, (3) selecting the studies, (4) charting the data (data extraction), and (5) collating, summarizing, and reporting the results. Furthermore, the PRISMA-ScR (Preferred Reporting Items for Systematic reviews and Meta-Analyses extension for Scoping Reviews; Tricco et al., 2018) was used (Supplementary Material 1).

### Search Strategy

Searches were conducted in the PubMed, Scopus, Medline, PsycINFO, and Web of Science databases from October 2020 to March 2021.

The search strategies combined key terms and Medical Search Headings (MESH) terms based on the patient problem (or population), intervention, comparison, or control, and outcome (PICO) framework as follows: (“OSAS” OR “Obstructive Sleep Apnea Syndrome”) AND (“CPAP” OR “Continuous Positive Airway Pressure”) AND (“Motivational intervention” OR “Motivational treatment” OR “Motivational interviewing”) AND (“Adherence” OR “Compliance” OR “CPAP use”) (Huang et al., 2006). Boolean and truncation operators were used to systematically combine searched terms and to list documents containing variations on search terms, respectively. The search syntax was modified as appropriate for each database.

### Inclusion and Exclusion Criteria

Only original articles that (1) employed randomized controlled trials (RCTs), non-randomized trials, or non-controlled trials study designs, (2) were published in English, and (3) examined the impact of motivational interventions on adherence (primary outcome) to CPAP use in adult (4) with a primary diagnosis of OSAS (5) were included. Records were excluded if they (1) considered only biomedical outcome variables, (2) were review articles, single-case studies, qualitative studies, mixed-method studies, protocol studies, or workplace interventions.

Unpublished works were not considered. No restrictions were set for the date of publication.

### Study Selection

Following the search and exclusion of duplicates, two reviewers (authors 1 and 2) independently assessed the eligibility of the articles first on the title and the abstract, and the full-text according to the inclusion criteria. Author 3 resolved disagreements. The reference lists of all selected articles and relevant systematic reviews (Weaver, 2019) were manually screened to identify any further references for possible inclusion—but none was found.

## Results

### Study Selection

A search of electronic databases identified 115 reports, of which 98 were excluded based on information from the title and abstract. The remaining 17 articles were evaluated for inclusion by reviewing their full text and resulted in the exclusion of 6 records for the following reasons: (1) focused on motivational App development and testing (*n* = 1; Alismail and Olfman, 2020), (2) aimed at validating a screening questionnaire (*n* = 1; Sawyer et al., 2014), (3) was a review (*n* = 1; Weaver, 2019) or (4) a protocol study (*n* = 1; Williams et al., 2014), (5) OSAS was not reported as the primary diagnosis (*n* = 1; Whittington et al., 2020) and (6) employed a mixed-method study design (*n* = 1; Broström et al., 2013). Eleven records finally were included in this review (Roecklein et al., 2010; Sparrow et al., 2010; Olsen et al., 2012; Aloia et al., 2013; Lai et al., 2014; Lo Bue et al., 2014; Dantas et al., 2015; Bakker et al., 2016; Jean-Louis et al., 2017; Pengo et al., 2018; Rudilla et al., 2021).

**Figure 1:**
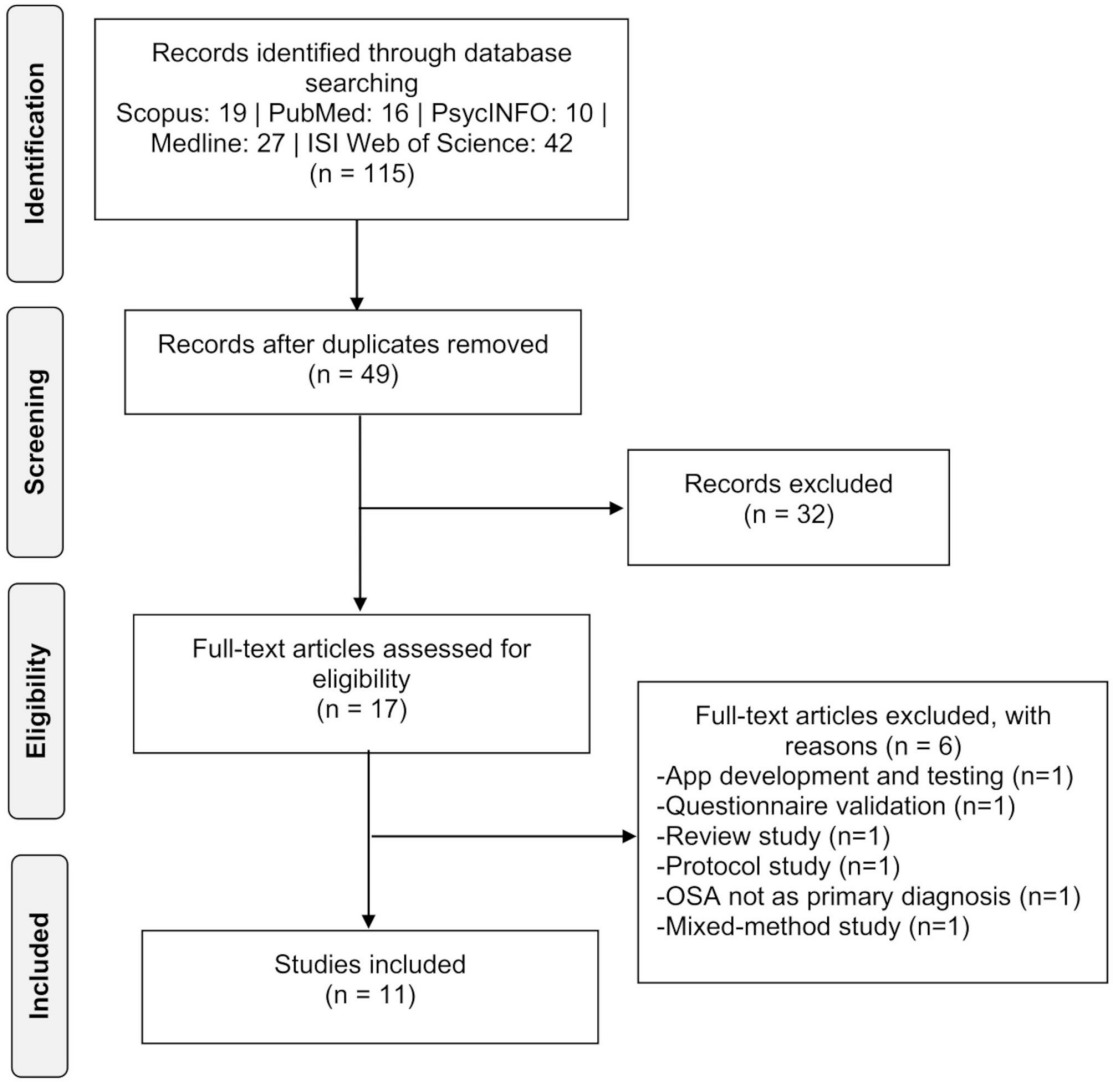
Flow chart diagram.

The flowchart presented in Figure 1 provides step-by-step details of the study selection.

### Study Characteristics

Details of the 11 included papers are provided in Table 1.

**Table 1:** Characteristics of the included studies.

**Author, year**	**Country**	Study aim^*^	**Sample size (*n*)**	**Age (yrs): *Mean (SD), range***	**Male gender: *n(%)***	**BMI (kg/m^2^): *Mean (SD); range***	**ESS**	**AHI: *events/h (SD)***	Follow-up points^**^	**Primary outcomes: *measure***	**Secondary outcomes: *measure***	**Drop-out *N (%)***	**Results (primary outcomes)^†^**	**Results (secondary outcomes)^†^**
Aloia et al. (2013)	US	To compare motivational enhancement intervention (*IG*) vs. *TAU* vs. educational group (*ED*) in improving **adherence** to CPAP, and **self-efficacy** and **decisional balance**	*N* = 227 *TAU* = 74 *ED* = 80 *IG* = 73	*TAU* = 52.4 (11.8) *ED* = 47.0 (11.4) *IG* = 51.7 (10.0)	*TAU* = 57 (77) *ED* = 48 (60) *IG* = 45 (62)	*TAU* = 35.8 (8.4) *ED* = 35.0 (7.3) *IG* = 35.1 (7.3)	*TAU* = 11.9 (5.1) *ED* = 12.6 (4.9) *IG* = 11.6 (5.2)	*TAU* = 48.2 (26.2) *ED* = 46.1 (23.2) *IG* = 45.7 (23.8)	**T0–**T1–T2–**T3–T6–T12**	Adherence to CPAP: Machine usage (h/night)	Self-efficacy and decisional balance: *ad hoc* measure	*TAU*: 25 (33) *ED*: 27 (33) *IG*: 26 (36)	Adherence declined over time for all three groups. Among moderate users (≥2 but <6 h/night) in the first week, average adherence at T12 in the *IG* was significantly higher (*M* = 4.12 h/night, *SE* = 0.42) than the average adherence in the other two control groups (*TAU*: *M* = 2.46 h/night, *SE* = 0.40; *ED*: *M* = 3.21 h/night, *SE* = 0.40; *p* = 0.002).	For self-efficacy at T12 *IG* had the highest mean confidence score for moderate PAP users and *ED* had the highest mean confidence score for high users, but there were no significant group differences. *TAU*: T0 = 20.96 (2.88); T3 = 20.61 (4.49); T6 = 21.00 (5.06); T12 = 19.83 (6.29) *ED*: T0: 21.11 (3.21); T3 = 21.05 (4.50); T6 = 21.04 (5.03); T12 = 20.98 (5.19) *IG*: T0 = 20.63 (3.26); T3 = 22.48 (3.08); T620.96 (4.23); T12 = 21.25 (4.84) For decisional balance the moderate user group (≥ 2 but <6 h/night) of *IG* demonstrated a higher index score at T12 when compared to the average of other two groups for moderate users (*p* = 0.04). *TAU*: T0 = 47.11 (6.02); T3 = 45.51 (7.67); T6 = 45.51 (7.90); T12 = 45.08 (10.55) *ED*: T0 = 46.32 (5.70); T3 = 46.33 (7.69); T6 = 44.66 (9.14); T12 = 45.76 (8.38) *IG*: T0 = 45.28 (6.94); T3 = 46.13 (6.23); T6 = 45.91 (6.95); T12 = 46.61 (7.47)
Bakker et al. (2016)	US	To compare motivational enhancement intervention (*IG*) vs. *TAU* in improving **adherence** to CPAP and **sleep duration**	*N* = 83 *TAU* = 42 *IG* = 41	*TAU* = 63.9 (7.4) *IG* = 63.8 (8.3)	*TAU* = 28 (66.7) *IG* = 27 (65.9)	*TAU* = 30.6 (4.5) *IG* =31.6 (5.9)	*TAU* = 7.7 (4.2) *IG* = 8.4 (4.8)	*TAU* = 23.7 (15.9, 31.4) *IG* = 21.8 (17.4, 31.0)	**T0**–T6–**T12**	Adherence to CPAP: Machine usage (h/night); % days of CPAP use	Sleep duration	*TAU*: 16 (38%) *IG*: 15(36%)	At T6, average nightly adherence was 99.0 min/night higher in *IG* compared with *TAU* (*p* = 0.003). At T12 a consistent difference in adherence between arms of 97 min/night (*p* = 0.006) favoring *IG*.	There were no significant differences in sleep duration, either over time within arms.
Dantas et al. (2015)	PT	To compare motivational interviewing (*IG*) vs. *TAU* vs. educational group (*ED*) in improving **adherence** to CPAP, **conviction, and confidence**, and **daytime sleepiness**	*N* = 61 *ED* = 20 *TAU* = 20 *IG* = 21	*ED* = 56.4 (8.5) *TAU* = 57.1 (10.6) *IG* = 56.2 (11.2)	*ED* = 18 (86) *TAU* = 16 (80) *IG* = 13 (65)	*ED* = 32.5 (5.0) *TAU* = 31.3 (4.6) *IG* = 34.8 (7.2)	NR	*ED* = Moderate ≥15: 5 (24); Severe > 30: 16 (76) *TAU* = Moderate ≥15: 5 (25); Severe >30: 15 (75) *IG* = Moderate ≥15: 6 (30); Severe >30: 14 (70)	T0–**T1–T2**	Adherence to CPAP: Machine usage (h/night); % days of CPAP use	AHI Daytime sleepiness: ESS Conviction: VAS 0–10 Confidence: VAS 0–10	*ED*: 1	The *IG* presented higher adherence to CPAP—percentage of days of use >4 h (89.8% *p* = 0.013), mean effective use per effective day (6.2; *p* = 0.000) at T2, compared with *TAU* and IN groups.	*IG* showed lower AHI (2.7; *p* = 0.019) at T2 when compared with the other two groups. For conviction, no differences were detected. Confidence was higher in the *IG* group at T2 than at T1 (*p* = 0.000). The ESS presented a significant reduction (*p* = 0.000) in the *IG* and the IN (*p* = 0.008) but was higher in the *TAU* (*p* = 0.015).
Jean-Louis et al. (2017)	US	To compare motivational-based intervention (*IG*) vs. *ED* in improving **adherence** to physician-recommended assessment	*N* = 380 *ED* = 143 *IG* = 160	*ED*: 57.9 (13.0) *IG*: 60.2 (13.6)	*ED* = 28.7% *IG* = 28.8%	*ED*: <25 = 14.9%; 25-30 = 21.3%; >30 = 63.8% *IG*: <25 = 10%; 25-30 = 21.2%; >30 = 68.8%	NR	NR	**T0 -** T6	Adherence to recommended OSAS evaluations: directly asked	NR	*ED*: 47 (25%) *IG*: 30 (16%)	No significant differences between the two arms regarding adherence to OSAS evaluation or treatment.	NR
Lai et al. (2014)	CH	To compare motivational enhancement intervention (*IG*) vs. *TAU* in improving adherence to CPAP, **self-efficacy, risk perception, outcome expectancies, daytime sleepiness**, and **quality of life**	*N* = 100 *TAU* = 51 *IG* = 49	*TAU*: 51 (10.0) *IG*: 53 (10.0)	*TAU* = 42 (82) *IG* = 41 (84),	*TAU* = 29.3 (5.4) *IG* = 28.6 (5.5)	*TAU* = 9 (5.0) *IG* = 9.5 (5.8)	*TAU* = 28.2 (20.3, 53.6) *IG* = 30.7 (20.6, 52.2)	T1w**–T1–T3**	Adherence to CPAP: Machine usage (h/night)	Risk perception and Expectancies: *ad hoc* measure Daytime sleepiness: ESS Self-efficacy: SEMSA Quality of life: FOSQ, CSAQLI, SF-36	*TAU*: 1 *IG*:1	The *IG* had better CPAP use (higher daily CPAP usage by 2 h/d) [Cohen *d* = 1.33, *p* < 0.001], a four-fold increase in the number using CPAP for ≥70% of days with ≥4 h/d (*p* < 0.001) compared with *TAU* at T3. *TAU*: T1w = 2.9 (2.5); T1 = 2.6 (2.3); T3 = 2.4 (2.3) *IG*: T1w = 5.5 (1.8); T1 = 4.8 (1.6); T3 = 4.4 (1.8)	No between-group differences on Risk perception, Outcome expectancies and in any score of the three health-related quality-of-life scales. The *IG* had greater improvements in ESS by 2.2 units (*p* < 0.001) and SEMSA by 0.2 units (*p* < 0.012) compared with *TAU*.
Lo Bue et al. (2014)	IT	To compare motivational enhancement intervention (*IG*) vs. *TAU* in improving adherence to CPAP and **daytime sleepiness**	*N* = 40 *TAU* = 20 *IG* = 20	*TAU*: 55.65(8.25) *IG*: 58.55 (13.2)	27	*TAU*: 34 (5.99) *IG*: 33.93 (5.44)	*TAU*: 10.55 (6.21) *IG*: 8.95 (5.74)	*TAU*: 44.45 (25.18) *IG*: 44.05 (16.90)	T0–T1–T3–T6–T12	Adherence to CPAP: Machine usage (h/night); % days of CPAP use	Daytime sleepiness: ESS	*TAU*: 2 *IG*: 1	During the first month, intervention group patients showed a higher number of nights with a device use ≥4 h. Average treatment adherence in the T1 (days of therapy with at least 4 h per night on the total number of days from device delivery) was 77.5% in *IG* and 55.7% in *TAU* (*p* = 0.022). No significant differences in other follow-up points.	Both in the *IG* and *TAU*, ESS was lower at the 3rd, 6th, and 12th month than at baseline with no significant differences between the two groups
Olsen et al. (2012)	AU	To compare motivational interviewing (*IG*) vs. educational intervention (*ED*) in improving acceptance and adherence to CPAP, **daytime sleepiness, risk perception, health-related quality of life, self-efficacy**, and **satisfaction with therapy**	*N* = 106 *ED* = 53 *IG* = 53	*ED*: 57.74 (9.51) *IG*: 55.14 (12.58)	*ED*: 38 (76) *IG*: 31 (62)	*ED*: 34.65 (7.07) *IG*: 34.28 (6.71)	*ED*: 11.14 (5.32) *IG*: 10.82 (4.41)	*ED*: 32.39 (20.32) *IG*: 36.23 (27.76)	**T1–T2–T3**–T12	Adherence to CPAP: Machine usage (h/night) Acceptance of CPAP: rejection rate	Daytime sleepiness: ESS Self-efficacy: SMSA Benefits: SMSA Risk perception: SMSA Health-related quality of life: FOSC Satisfaction with Therapy and Therapist: STTS-R	NR	Adherence declined over time in the two groups. The number of hours of CPAP use per night in the *IG* at T1, T2, and T3 was significantly higher compared with *TAU* (*p* < 0.005). No significant between-group difference at T12. *TAU*: T1 = 3.25 (2.83); T2 = 3.22 (2.76); T3 = 3.16 (2.69); T12 = 3.00 (3.18) *IG*: T1= 4.85 (2.55); T2 = 4.73 (2.62); T3 = 4.63 (2.69); T12 = 4.21 (3.25) Significantly more participants in the *IG*	For ESS, no between-group differences, but the main effect for time emerged (TI–T3 mean ESS: 10.04 vs. 6.73, *p* < 0.01; T2–T3 mean ESS: 8.90 vs. 6.73, *p* < 0.01). For SMSA, *IG* showed greater self-efficacy than *TAU* but not significantly (3.42 vs. 3.11). A significant main effect for time is reported for the reduction in risk perception between T1 and T3 (*p* < 0.05) in all patients. A significant main effect for time is reported for better health-related quality of life from T1 to T2, from T2 to T3, and from T1 to T3 (all *p* < 0.01).
													commenced on CPAP at T3 (6% rejection rate) compared to *ED* (28% rejection rate) (*p* = 0.004). At T12, the difference between CPAP commencement in the *IG* (4% rejection rate) compared to the *ED* (26% rejection rate) was still significant, (*p* = 0.002).	The STTS-R was not correlated with adherence at any of the three follow-up points.
Pengo et al. (2018)	UK	To compare positive (*IG*+) and negative framed messages (*IG*–) based on motivational strategies and *TAU* in improving **adherence** to CPAP and **daytime sleepiness**	*N* = 112 *TAU* = 36 *IG*(+) = 36 *IG*(–) = 37	*TAU*: 53.5 (12.5) *IG*(+): 46.7 (12.2) *IG*(–): 47.1 (11.7)	*TAU*: 31 *IG*(+):25 *IG*(–):28	*TAU*: 37.3 (11.7) *IG*(+):36.0 (8.3) *IG*(–):36.3 (7.6)	*TAU*: 11.9 (6.1) *IG*(+): 10.8 (5.3) *IG*(–): 11.2 (6.2)	NR	T2w–T6w	Adherence to CPAP: Machine usage (total hours)	Daytime sleepiness: ESS	*TAU*: 11 (30.5) *IG*(+): 5 (14%) *IG*(–): 8 (21.63%)	The *IG*(+) showed significantly greater CPAP usage after 2 weeks (total use 53.7 ± 31.4 h) compared to the *IG*(–) (35.6 ± 27.4) and *TAU* (40.8 ± 33.5 h, *p* < 0.05); however, no differences were seen at 6 weeks.	ESS improved in all patients (baseline 11.0 (6.0) vs. ESS at 2 weeks 9.2 (5.9) points, *p* < 0.0001) with no significant differences among groups. There was no difference between 2 and 6 weeks.
Roecklein et al. (2010)	US	To compare motivational enhancement intervention (*IG*) vs. *TAU* in improving **adherence to CPAP**, apnea, **side effects of CPAP use, daytime sleepiness**	*N* = 30 *TAU* = 16 *IG* = 14	*TAU*: 46.10 (11.50) *IG*: 46.60 (11.30)	*TAU*: 25% *IG*: 30%	*TAU*: 42.18 (7.61) *IG*: 42.06 (8.91)	*TAU*: 11.25 (4.15) *IG*: 11.92 (5.50)	*TAU*: 45.82 (42.38) *IG*: 42.69 (34.34)	**T2w**–T3	Adherence to CPAP: Machine usage (h/night) Self-reported CPAP usage: CPAP/BiPAP Questionnaire	Side effects of CPAP use: SEQ Apnea: CSAQLI Daytime sleepiness: ESS Readiness, Motivation, Knowledge and Social support: *ad hoc* measure	*TAU*:1 *IG*:1	There was no difference in objective average daily use or total hours between-group at T2w and T3, but in both groups, the daily use of CPAP decreased. Individuals in the *IG* with self-report measure reported using CPAP longer than controls in both time points, but at T3 the adherence-reported level is significantly higher in *IG* [*F*_(1, 22)_ = 7.13, *p* < 0.05] *TAU*: T2w = 7.11 (8.45); T3 = 66.43 (32.07) *IG*: T2w = 11.26 (7.89); T3 = 93.75 (15.83)	Groups did not differ in rates of CPAP side effects, daytime sleepiness, or symptoms between time points. There was a main effect of time on symptoms due to the expected decrease in symptoms over time [*F*_(1, 25)_ = 15.49, *p* < 0.01]. Readiness at T2w predicted total therapy hours at T3 (β = −207.97, Seβ = 94.60, *p* < 0.05), readiness improved significantly over time [*F*_(1, 25)_ = 49.44, *p* < 0.05].
Rudilla et al. (2021)	SP	To compare motivational interviewing (*IG*) vs. *TAU* in improving **adherence** to CPAP, **motivation, perceived competence, quality of life, daytime sleepiness, emotional state**, and **social relations**	*N* = 83 *TAU* = 42 *IG* = 41	*TAU*: 57.51 (12.19) *IG*: 61.35 (13.11)	*TAU*: 28 *IG*: 32	NR	*TAU*: 13.02 (4.29) *IG*: 10.35 (4.53)	*TAU*: 49.95 (17.85) *IG*: 46.30 (18.80)	**T1–T3**	Adherence to CPAP: Machine usage (h/night)	Motivation: open-ended questions Perceived competence: CEPCA Quality of life: VAWBS-A Daytime sleepiness: ESS Emotional state, Activities, and social relations: *ad hoc* measure	*TAU*: 2 *IG*: 1	For CPAP adherence, statistically significant results were obtained in favor of *IG* (*p* < 0.01), with a mean difference of 1.60 h (95% CI, 0.60–2.61).	CEPCA was significantly higher in *IG* at T3 with a mean difference of 4.61 (95% CI, 3.49 to 5.72) (*p* < 0.001). VAWBS-A was significantly higher in *IG* at T3 (*p* < 0.001.). No statistically significant differences were observed for ESS when comparing the before-after change between the treatment arms at T3, but there were statistically significant differences found when comparing the outcomes between the two study groups.
Sparrow et al. (2010)	US	To compare motivational enhancement intervention (*IG*) vs. educational group (*ED*) in improving **adherence** to CPAP, **daytime sleepiness, sleep-related symptoms, depression, behavioral alertness, CPAP self-efficacy**, and **decisional balance**	*N* = 250 *ED* = 126 *IG* = 124	*ED*: 54.0 (45.0-62.0) *IG*: 56.0 (48.0-63.0)	*ED*: 105 (83.3) *IG*: 100 (80.7)	*ED*: 35.9; 31.9-42.1 *IG*: 34.4; 30.1-40.1	*ED*: 11.0 (8.0-15.0) *IG*: 10.0 (6.0-15.0)	*ED*: 40.5 (21.0-64.0) *IG*: 36.0 (22.0-63.0)	T0**–T6–T12**	Adherence to CPAP: Machine usage (h/night)	Daytime sleepiness: FOSQ Sleep-related symptoms: SSC Depression: CES-D Behavioral alertness: PVT CPAP Self-Efficacy: *ad hoc* measure CPAP Decisional Balance: *ad hoc* measure	*ED*: 14 *IG*: 12	The intervention had a significant effect on CPAP adherence: median observed CPAP use in patients in *IG* was approximately 1 h/night higher than in subjects of *TAU* at 6 months (*p* = 0.006) and 2 h/night higher at 12 months (*p* = 0.004)	CPAP adherence was significantly associated with a greater reduction in sleep apnea symptoms and depressive symptoms and a greater improvement in functional status. At T6 and T12 *IG* scored significantly higher than *TAU* on self-efficacy and decisional balance *TAU*(self-efficacy): T6 = 4.2, 3.0–4.8; T12 = 4.2, 3.0–5.0 *IG*(self-efficacy): T6 =4.4, 3.8–5.0; T12 = 4.6, 3.6–5.0 *TAU*(decisional balance): T6 = −3.0, −10.5–4.0; T12 = −2.0, −11.0–4.0 *IG*(decisional balance): T6 = 1.2, −6.0–7.0; T12 = 0.0, −6.0–6.0

*AHI, Apnea-Hypopnea index; AU, Australia; CEPCA, Questionnaire of Evaluation of Perceived Competence in Adherence to CPAP in OSAS; CES-D, the Center for Epidemiological Studies Depression; CH, China; CSAQLI, Calgary Sleep Apnea Quality of Life Index; ESS, Epworth Sleepiness Scale; FOSQ, Functional Outcomes of Sleep Questionnaire; IT, Italy; PT, Portugal; PVT, Psychomotor Vigilance Task; SEMSA, Self-Efficacy Measures Of Sleep Apnea; SEQ, The Side Effects Questionnaire; SF-36, Short Form-36 Health Survey Questionnaire; SMSA, Self-Efficacy Measure for Sleep Apnea; SP, Spain; SSC, Sleep Symptoms Checklist; STTS-R, The Satisfaction with Therapy and Therapist Scale–Revised; UK, The United Kingdom; US, The United States; VAS, Visual Analog Scale; VAWBS-A, Visual Analogical Well-Being Scale for Apnoea*.

* *Patient-Reported Outcomes in bold;*

** *Psychological data measurements in bold. Where not otherwise specified, times are expressed in months*.

† *Only significant p-values were reported. NR, not reported*.

The selected articles were published from 2010 (Roecklein et al., 2010; Sparrow et al., 2010) to 2021 (Rudilla et al., 2021), and were conducted in the USA (Roecklein et al., 2010; Sparrow et al., 2010; Aloia et al., 2013; Bakker et al., 2016; Jean-Louis et al., 2017; *n* = 5), China (*n* = 1; Lai et al., 2014), Australia (*n* = 1; Olsen et al., 2012), UK (*n* = 1; Pengo et al., 2018), Portugal (*n* = 1; Dantas et al., 2015), Spain (*n* = 1; Rudilla et al., 2021) and Italy (*n* = 1; Lo Bue et al., 2014). All studies employed a randomized parallel-group trial design.

### Description of Participants

Selected contributions included a total of 1472 adult participants of both genders (age range: 34-75; mean age = 54.66 years) with a diagnosis of OSAS at their first use of a recognized sleep diagnostic tool with an Oxygen Desaturation Index (ODI) of ≥ 5 per hour or an Apnea-Hypopnea Index (AHI) ≥ 5 per hour.

### Description of Intervention

**Figure 2:**
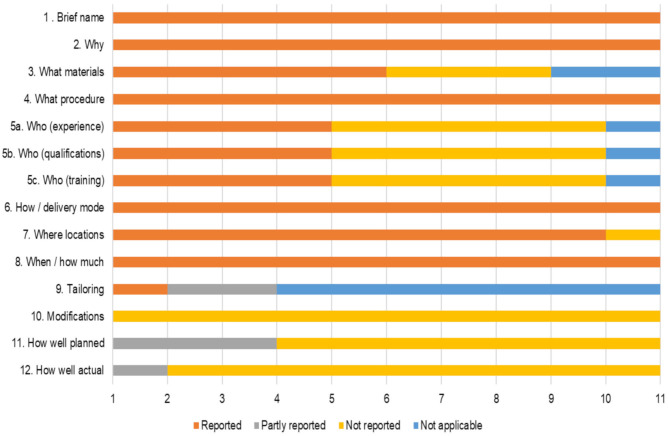
Proportion of TIDieR items not/partly or fully included in each of the 11 trial reports.

The main characteristics of the interventions are reported in Supplementary Material 2 using the CONsolidated Standards of Reporting Trials 2010 (Consort10) checklist (Schulz et al., 2010) and Supplementary Material 3 using the Template for Intervention Description and Replication (TIDieR) checklist (Hoffmann et al., 2014). Figure 2 showed substantial differences in reporting frequency occurred between TIDieR items in each of the 11 trial reports.

#### Intervention Group

Intervention groups were based on the principles and techniques of Motivational Interviewing (MI) (Olsen et al., 2012; Dantas et al., 2015; Rudilla et al., 2021) and Motivational Enhancement Therapy (MET) (Roecklein et al., 2010; Sparrow et al., 2010; Aloia et al., 2013; Lai et al., 2014; Lo Bue et al., 2014; Bakker et al., 2016), which combines MI with personalized assessments, feedback, and change plans (Miller, 1992).

The number of intervention sessions ranged from one (Roecklein et al., 2010; Lai et al., 2014; Lo Bue et al., 2014; Dantas et al., 2015) to 16 (Sparrow et al., 2010), and the length of each session varied from 2 (Pengo et al., 2018) to 90 min (Rudilla et al., 2021).

In all the selected studies, the treatment consisted of providing OSAS-related information, addressing treatment expectations, and ambivalence toward the use of the CPAP, as well as in defining goals and motivating treatment adherence. The individual's understanding of the health risks associated with untreated OSAS and the extent to which they believed that consistent CPAP use would lead to symptoms improvement were also discussed during treatment.

However, only 4 out of 11 contributions detailed the motivational strategies employed in the intervention (Olsen et al., 2012; Aloia et al., 2013; Jean-Louis et al., 2017; Rudilla et al., 2021).

In the study by Aloia et al. (2013) and Jean-Louis et al. (2017), the practitioners first discussed with the patients what they already knew or were interested in learning about the impact that sleep apneas and the use of the CPAP have on health and asked the patients for permission to provide information. Information was given in a neutral, non-judgmental fashion to elicit the patient's interpretation (the elicit-provide-elicit technique). The goal-setting technique was then used to identify and set realistic goals that align with the patients' values (Aloia et al., 2013; Jean-Louis et al., 2017). Moreover, in the study by Jean-Louis et al. (2017), coaching and role-playing techniques were applied. In the final phase of the intervention, the practitioners also complimented the patients for the achieved results and offered further education on the benefits of CPAP therapy in case resistance to change was encounter.

Instead, in the study by Olsen et al. (2012), the first session focused on increasing readiness to change by exploring the patients' motivation to treatment, assessing discrepancies between their ideal and current behavior (decisional balance technique), and eliciting self-motivational statements (change talk). The importance of CPAP use was also discussed, and emphasis was placed on the patients' autonomy. The second session was aimed at strengthening the patient's commitment to change, summarizing the pros and cons of the use of CPAP, and setting realistic goals to achieve. Following, the patient's improvements were reviewed and reinforced, while key barriers to CPAP use were identified and addressed. In the study by Rudilla et al. (2021), motivational strategies were adapted to the patient's stage of change (Prochaska and DiClemente, 1983). When in the pre-contemplation phase, information was provided while dealing with resistance to change and supporting the patients' self-efficacy. In the contemplation phase, the pros and cons of change were discussed to support goal setting. In the determination phase, emphasis was made on setting a change plan. In the maintenance phase, strategies to deal with risk situations were provided. In case of relapses, the patients were helped to understand their reason causes, while confidence in their ability to make behavioral changes and related action plans were further supported. Further, in the study by Pengo et al. (2018), health-related information was offered in terms of either risk or benefits framed via positive or negative message.

Notably, only 4 out of 11 studies (Aloia et al., 2013; Dantas et al., 2015; Jean-Louis et al., 2017; Rudilla et al., 2021) tailored motivational intervention strategies according to the patient's initial degree of readiness to change and confidence in their ability to succeed.

In four studies the treatment was conducted by a motivational-trained-nurse (Olsen et al., 2012; Aloia et al., 2013; Lai et al., 2014; Rudilla et al., 2021), in two studies by a sleep doctor/sleep technician (Lo Bue et al., 2014; Pengo et al., 2018), and in one record by a psychologist (Bakker et al., 2016) and by a trained health educator (Jean-Louis et al., 2017), respectively. A multidisciplinary intervention involving a pulmonologist, psychologist, and physiotherapist was employed in one study (Dantas et al., 2015), while Sparrow et al. (2010) implemented the intervention with the use of an automated telephone-linked communication system. Only Roecklein et al. (2010) did not mention who conducted the intervention.

The treatment was conducted through regular in-person meetings in four studies (Roecklein et al., 2010; Olsen et al., 2012; Aloia et al., 2013; Rudilla et al., 2021), while in five contributions (Sparrow et al., 2010; Lo Bue et al., 2014; Jean-Louis et al., 2017) motivational interventions were entirely provided via remote interactions, and in three studies the interventions began in-person followed by telephone-based follow-ups sessions (Lai et al., 2014; Bakker et al., 2016; Pengo et al., 2018) or the use of an App motivating and assisting the use of the CPAP.

*Ad hoc* video education offering real-life experiences with the CPAP was also used in 2 out of 11 studies in addition to the motivational intervention (Lai et al., 2014; Bakker et al., 2016). Moreover, a single motivational group-format intervention with 20 participants was employed in one contribution (Dantas et al., 2015).

The theoretical background of the intervention was reported in 5 out of 11 studies (Roecklein et al., 2010; Sparrow et al., 2010; Olsen et al., 2012; Aloia et al., 2013; Rudilla et al., 2021). Aloia et al. (2013) and Roecklein et al. (2010) referred both to the Social Cognitive Theory (SCT; Bandura et al., 1999) and the Transtheoretical Model of Change (TTM; Prochaska and DiClemente, 1983). In Rudilla et al. (2021) and Sparrow et al. (2010), the interventions were respectively based on the TTM and SCT, while in Olsen et al. (2012) on the Health Belief Model (HBM; Becker, 1974).

#### Control Group

Six studies compared the intervention group with a standard care condition only (treatment as usual; *TAU*) (Roecklein et al., 2010; Lai et al., 2014; Lo Bue et al., 2014; Bakker et al., 2016; Pengo et al., 2018; Rudilla et al., 2021). Educational sessions focused on increasing awareness on the benefits of a healthy lifestyle and of the use of the CPAP were, instead, used as controls in two contributions (Sparrow et al., 2010; Olsen et al., 2012; Aloia et al., 2013; Jean-Louis et al., 2017), while two records included both *TAU* and educational controls (Aloia et al., 2013; Dantas et al., 2015).

### Effects of the Intervention Across Time-Points

#### Primary Outcomes

Study duration ranged from 2 weeks (Pengo et al., 2018) to 12 months (Sparrow et al., 2010; Olsen et al., 2012; Aloia et al., 2013; Lo Bue et al., 2014; Bakker et al., 2016). In one study, the intervention had a duration of 2 months (Dantas et al., 2015), 3 months (Roecklein et al., 2010; Lai et al., 2014; Rudilla et al., 2021), or 6 months (Jean-Louis et al., 2017), respectively.

In 10 studies data related to hourly CPAP usage were used to measure adherence to treatment as a primary outcome (Roecklein et al., 2010; Sparrow et al., 2010; Olsen et al., 2012; Aloia et al., 2013; Lai et al., 2014; Lo Bue et al., 2014; Dantas et al., 2015; Bakker et al., 2016; Pengo et al., 2018; Rudilla et al., 2021), and in 1 study patients were directly asked if they were following the indications (Jean-Louis et al., 2017). Only Roecklein et al. (2010) used both objective (CPAP usage data) and subjective (self-report *ad-hoc* questionnaire) measures of adherence.

Nine out of 11 studies showed that motivational interventions were effective in increasing the average hours of CPAP use (Sparrow et al., 2010; Olsen et al., 2012; Aloia et al., 2013; Lai et al., 2014; Lo Bue et al., 2014; Dantas et al., 2015; Bakker et al., 2016; Pengo et al., 2018; Rudilla et al., 2021). In particular, significantly higher CPAP adherence was found after 2 weeks (Pengo et al., 2018) and 1 month (Lo Bue et al., 2014) from CPAP titration among participants in the motivational group compared with those receiving usual care, despite in Lo Bue et al. (2014) results were not maintained over time. Similarly, in the study by Dantas et al. (2015), patients assigned to the experimental condition presented significantly higher adherence to CPAP after 2-month from CPAP titration than those in the *TAU* and educational controls. Moreover, three studies assessed adherence to CPAP after 3 months from the beginning of the treatment and showed that motivational interventions were significantly more effective than *TAU* (Olsen et al., 2012; Lai et al., 2014; Rudilla et al., 2021) and educational controls (Olsen et al., 2012), respectively. Participants in the motivational interventions also revealed a significantly higher CPAP adherence compared with *TAU* (Bakker et al., 2016) and the educational control group (Sparrow et al., 2010) at 6- and 12-month follow-ups in 2 records and another study showed significant between-group differences at 12 months in favor of those participants in the motivational group who displayed moderate levels of adherence during their first week of CPAP use compared to both *TAU* and educational controls (Aloia et al., 2013). Instead, no significant between-group differences were found by Jean-Louis et al. (2017) and by Roecklein et al. (2010)—but a greater likelihood of adhering to CPAP was detected among patients receiving the motivational intervention compared to those participating in the educational group. Specifically, in Roecklein et al. (2010), significantly higher self-reported adherence was reported in the intervention group at 2-week and 3-month follow-ups.

**Figure 3:**
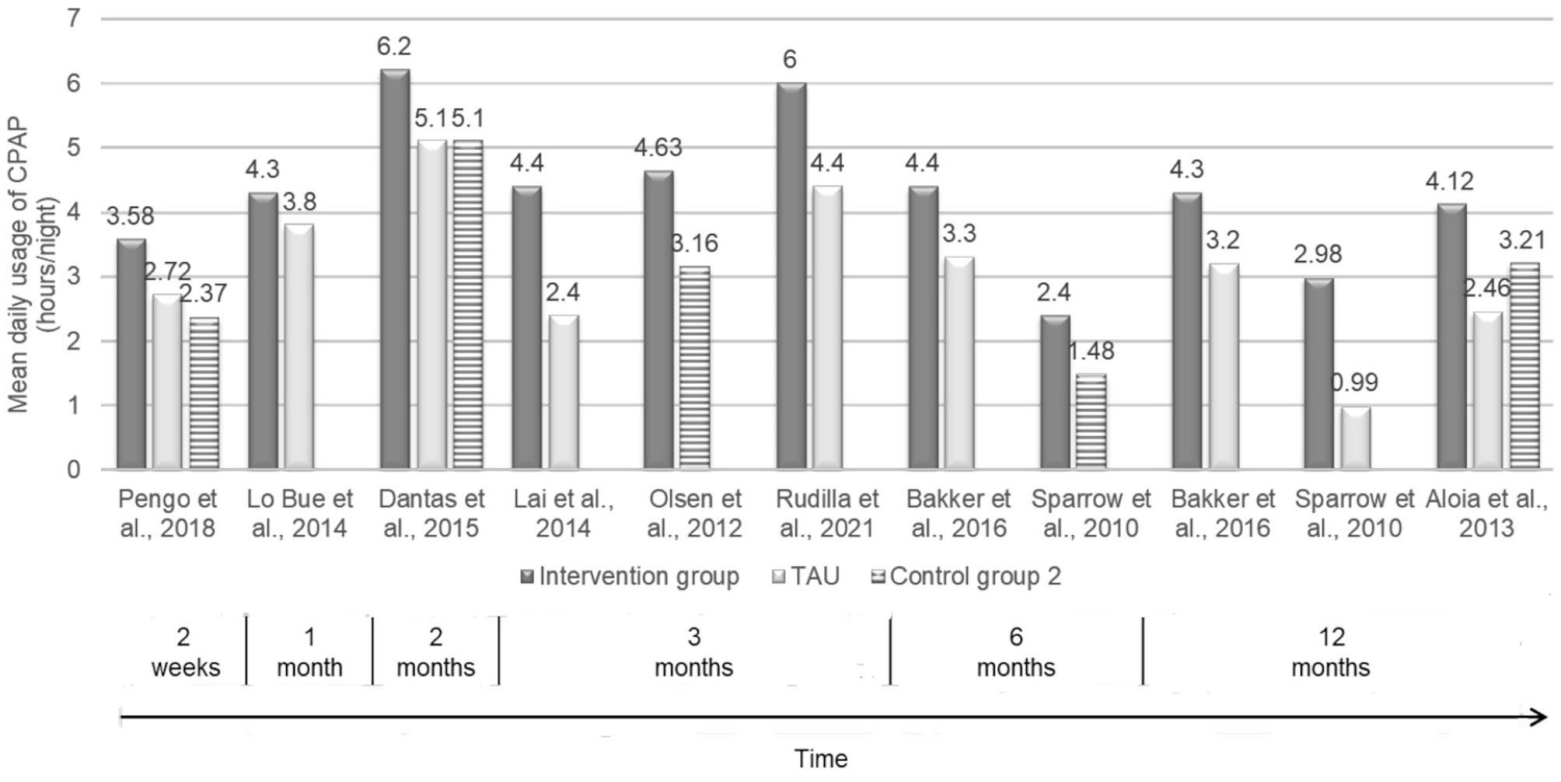
Mean daily usage of CPAP (hours/night) across conditions at different time-points.

**Figure 4:**
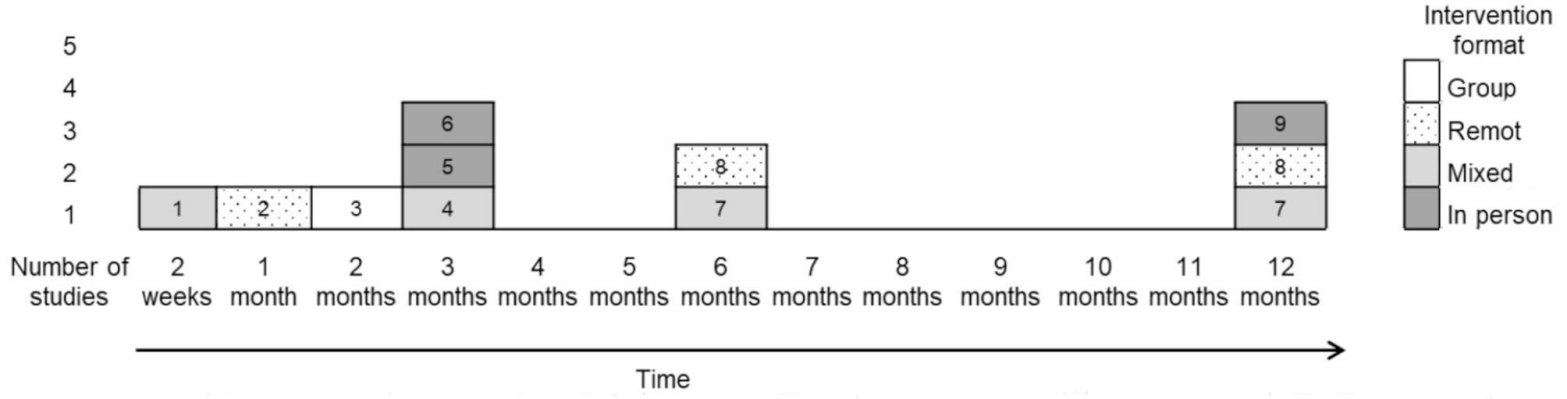
Efficacy of motivational interventions by type of intervention format. 1, Pengo et al., 2018; 2, Lo Bue et al., 2014; 3, Dantas et al., 2015; 4, Lai et al., 2014; 5, Olsen et al., 2012; 6, Rudilla et al., 2021; 7, Bakker et al., 2016; 8, Sparrow et al., 2010; 9, Aloia et al., 2013.

The figures below show the mean daily usage of CPAP (h/night) across conditions at different time-points as reported in the selected studies (Figure 3) and provide a summary of motivational interventions by type of intervention format that has been shown effective in increasing adherence to CPAP in the short- and long-term, according to Webb et al.' taxonomy (Webb et al., 2010) (Figure 4). The studies by Jean-Louis et al. (2017) and by Roecklein et al. (2010) were, therefore, excluded despite showing that patients in the intervention group had a greater likelihood of adhering to recommended CPAP compared with the educational group, and indicated significantly higher—but self-reported—adherence to CPAP than *TAU* and educational controls, respectively.

#### Secondary Outcomes

Seven out of the 11 selected studies made use of the Epworth Sleepiness Scale (ESS) to report the presence and intensity of daytime sleepiness (Roecklein et al., 2010; Olsen et al., 2012; Lai et al., 2014; Lo Bue et al., 2014; Dantas et al., 2015; Pengo et al., 2018; Rudilla et al., 2021).

In one study, after 3 months among participants in the motivational group compared with *TAU*. the ESS scores decreased significantly in the intervention group compared with *TAU* and educational controls at 1 and 2 months after CPAP titration (Dantas et al., 2015), while in another record (Lai et al., 2014) daytime sleepiness reduced significantly.

Instead, five studies failed to detect significant between-group differences in the ESS scores across conditions (Roecklein et al., 2010; Olsen et al., 2012; Lo Bue et al., 2014; Pengo et al., 2018; Rudilla et al., 2021), but an overall improvement of ESS among all the respondents was found in three studies (Olsen et al., 2012; Lo Bue et al., 2014; Pengo et al., 2018).

Moreover, one contribution showed over time an AHI improvement across conditions, and significantly lower AHI scores were registered after 2 months from CPAP titration in the motivational group compared with the educational and *TAU* controls (Dantas et al., 2015).

Two records (Olsen et al., 2012; Lai et al., 2014) also assessed OSAS risk perception. Although no significant between-group difference was found, it reduced significantly between 1 and 3-month follow-ups in one contribution (Olsen et al., 2012), while no significant improvement was shown by Lai et al. (2014).

Self-efficacy was assessed in six studies (Sparrow et al., 2010; Olsen et al., 2012; Aloia et al., 2013; Lai et al., 2014; Dantas et al., 2015; Rudilla et al., 2021) using: self-statements investigating the participants' confidence in their ability to follow the treatment recommendations (Sparrow et al., 2010; Aloia et al., 2013), the Self-efficacy Measure for Sleep Apnea (SMSA) (Olsen et al., 2012; Lai et al., 2014) the Questionnaire of Evaluation of Perceived Competence in Adherence to CPAP in OSA (Rudilla et al., 2021) or a Visual Analog Scale (VAS) (Dantas et al., 2015).

No between-group differences were detected with the first measure. However, self-efficacy levels decreased over time in the control conditions, while improving among participants in the motivational intervention in one study (Aloia et al., 2013). Moreover, Sparrow et al. (2010) observed a significant increase in self-efficacy at 6- and 12-month follow-ups in favor of the motivational group compared with *TAU*.

Significantly higher rates of self-efficacy were also found by Dantas et al. (2015) at 2-month follow-up among patients assigned to the motivational intervention compared with those receiving education. Self-efficacy increased in the motivational group and decreased in the control conditions.

Instead, even though higher SMSA scores were detected at 3-month follow-up in the motivational group compared with *TAU*, statistical between-group differences were found only in one contribution (Lai et al., 2014). These results were parallel to those by Rudilla et al. (2021), which showed that competence increased significantly at 3-month follow-up in the motivational group compared with *TAU* (Rudilla et al., 2021).

The construct of health-related quality was measured with the Functional Outcomes of Sleep Questionnaire (FOSC) in two contributions (Olsen et al., 2012; Lai et al., 2014). No between-group differences were observed in both studies, but in Olsen et al. (2012), increased scores were observed at 3-month follow-up across conditions. Instead, in the study by Rudilla et al. (2021) patients assigned to the motivational intervention showed better quality of life measured by the Visual Analogical Well-being Scale for apnoea than *TAU* after 3 months from CPAP titration.

Notably, the patients' readiness to change was investigated only in one study (Roecklein et al., 2010) using the SCT and the TTM Questionnaires adapted for CPAP. Results showed that motivation improved significantly over time both in the experimental and the educational control groups, and that baseline levels of readiness to change were negatively associated with CPAP use (h/night) at 3-month follow-up.

Potential side effects associated with the use of the CPAP were also explored by Roecklein et al. (2010), but no between-group differences were found across time points in both the motivational intervention and the educational control.

No significant between-group differences were also observed in the emotional state, daily activities, and social relationships of the participants in one study (Rudilla et al., 2021). Moreover, satisfaction with the therapy and the therapist did not appear to be related to CPAP adherence in another contribution (Olsen et al., 2012).

## Discussion

To our knowledge, this is the first scoping review of the literature aimed to explore the characteristics and impact of motivational interventions to promote adherence to CPAP therapy in patients with OSAS, commonly operationalized as increased daily hours of CPAP usage.

Results from 9 out of the 11 included studies showed that motivational interventions were more effective than usual care and/or educational programs in increasing adherence to CPAP. However, significant between-group differences favoring motivational interventions were mostly observed in the short term, and results were not always maintained over time. Moreover, adherence declined over time in both the motivational and control groups in six studies (Roecklein et al., 2010; Olsen et al., 2012; Aloia et al., 2013; Lai et al., 2014; Lo Bue et al., 2014; Bakker et al., 2016).

This outcome variability may to some extent be explained by the characteristics of the intervention, as some contributions reported on the effect of only one encounter, some of them had follow-up periods shorter than 3 months, and the treatment was delivered in different ways (i.e., in-person meetings or remote interactions; individual or group format).

Still, positive outcomes were observed even in brief motivational encounters of only 2 min (Pengo et al., 2018), and more than one encounter with a patient seems to increase the likelihood of an effect.

It should also be considered that aspects such as different types of healthcare professionals delivering the treatment, and their training and experience in the use of motivational strategies may have influenced the magnitude of the treatment, even if this cannot be shown in this review. Six out of 11 studies (Olsen et al., 2012; Aloia et al., 2013; Lai et al., 2014; Bakker et al., 2016; Jean-Louis et al., 2017; Rudilla et al., 2021) reported on how practitioners were trained, but only two of them (Lai et al., 2014; Bakker et al., 2016) assessed the treatment fidelity.

Most of the contributions also lack adequate details on the training of professionals, the contents of the interventions, and the theoretical models on which they were based.

Motivational interventions are of proven efficacy in improving adherence to treatment in patients suffering from various chronic conditions (Burke et al., 2003; Van Nes and Sawatzky, 2010; Maissi et al., 2011; Pietrabissa et al., 2012, 2015, 2017; Bonde et al., 2014; Soderlund, 2018)—but for the development of advanced intervention protocols, studies should include a more comprehensive description and assessment of the communicational strategies employed in the intervention. In fact, evidence exists for the correlation between poor compliance and health care providers' lack of communication skills. Often, patients deliberately ignore professionals' recommendations, even when change is needed, but this paradoxical behavior cannot be overcome with rational explanations. Therefore, mastering communication abilities in the medical setting is essential to promote low-cost interventions with a positive cost/benefit ratio.

Since motivational interventions largely depend on “listening” to the patients and accommodating their ambivalence and resistance to change—rather than “telling” and educating—the use of respectful, and compassionate communication may play a crucial role during the process of ending risk behaviors and/or adopting positive health behaviors in the clinical context.

In all the selected studies, the motivational strategies employed in the interventions were largely aimed to address treatment expectations and ambivalence toward the use of the CPAP, to define goals, and increase patients' confidence in their ability to make enduring behavioral change.

However, a relevant consideration is that only a few contributions targeted the interventions on the individuals' initial level of readiness and confidence to change, thus preventing from drawing valid conclusions over their outcomes.

Well-established theories of change (Prochaska and DiClemente, 1983; Miller and Rollnick, 1991) postulates that patients who are less motivated are expected to be more responsive to an intervention focused on increasing and maintaining motivation to change and that lower levels of self-efficacy reflect the number of previous failed attempts to make a change. Moreover, studies show that how patients with OSAS experience their first month of CPAP therapy may influence their long-term adherence to the device (Budhiraja et al., 2007; Collen et al., 2009; Perger et al., 2019).

In the selected contributions, participants were all first-time CPAP users. It is, therefore, reasonable to believe that the patients did not fall in the “resistant to change” category—for which motivational approaches are proven to be most effective. Accordingly, the only record that assessed the individuals' readiness to change over time (Roecklein et al., 2010) revealed a negative association between self-reported motivation to change and actual use of the CPAP in patients with OSAS. The reduced superiority of the motivational interventions in increasing adherence to CPAP among naïve uses might, therefore, also depend on the patients' level of motivation. These findings support the assumption that motivational intervention might even be counterproductive for highly motivated individuals (Resnicow and McMaster, 2012). Behavioral change is a complex phenomenon with multiple determinants that also includes psychological, motivational, and socio-environmental aspects. Therefore, assessing the individuals' adherence to a treatment regimen also means considering their level of problem awareness (reasons for change), willingness to change, and perceived ability to do so (Ceccarini et al., 2015). Subjective measures of adherence had relatively little representation in the reviewed studies. Yet, self-report indices of motivation to change do not necessarily equate to actual change in response to treatment, and they should be recognized as the patient's intent at that moment to change rather than a predictor for any real change in behavior.

Findings from this review also reveal that research testing the impact of motivational interventions on adherence to CPAP use among people suffering from OSAS is only recent—as selected studies were published between 2010 and 2021. Further studies need to be re-examined by including both objective and subjective measures of adherence, and longer follow-up periods that make sure the absence of any dissonance between the patients' intention to change and their current status.

### Strengths and Limitations

The results of the present scoping review should be interpreted with the following limitations in mind. First, the search of electronic databases was limited to trials published in the English language, and this may have led to the exclusion of relevant records. Second, there are limitations inherent in the decision not to include the gray literature, which may have further impacted the selection of studies and results. Third, this review investigated treatment components in isolation. As more trials are published, it may be useful to explore whether different effects are obtaining by combining treatment strategies, rather than investigating components in isolation. Fourth, inconsistent operationalizations and considerable variability in measures of adherence to treatment, the short-term assessment of outcomes, and the need for well-trained providers of motivational interventions were identified as major barriers to research progress in this area. As a result—despite the strengths of its well-defined methodology, careful selection of participants, extensive measures of psychological profile, and outcomes—this review has limited ability to project the likelihood of any adherence to CPAP being maintained over time following motivational interventions.

### Future Research and Practical Directions

Although motivational interventions are strongly recommended in clinical settings (Lim et al., 2019) to facilitate health behavior change in patients with pulmonary diseases (Minassian and Doran, 2020), the present findings suggest that several aspects may impact the intervention efficacy. More research providing a detailed description of motivational strategies and testing of their association with positive treatment outcomes is therefore needed. It would make adherence assessment more straightforward and increase knowledge on effective mechanisms of change.

Future research may also wish to apply both direct and indirect measures of adherence and to examine whether the duration of motivational intervention and frequency of sessions are associated with treatment effects. Moreover, qualitative investigations into the type of information or strategy patients with OSAS find most meaningful may aid in optimizing the content of the interventions.

Motivational interventions appear to be a useful strategy and that can easily be broadly disseminated, but more longitudinal study should test their longer-term effects.

Research also shows that motivational interventions can effectively be provided digitally to patients with OSAS using CPAP (Hu et al., 2021), with proven advantages and reduced costs (Appel et al., 2011; Bus et al., 2018). Further investigations should focus on the use of the different format of delivering motivational interventions including the use of new technologies, or the provision of motivational interventions in group settings (Velasquez et al., 2006; Channon et al., 2007; Tucker et al., 2017)—as the group format requires specific competence and additional tasks, and the motivational strategies are far more complex to operate than in individual sessions (Major and Palmer, 2001; Britt et al., 2004; Pietrabissa, 2018). This further supports the need for studies assessing treatment fidelity, and that also carefully describe the training offered to providers and the processes of supervision of motivational sessions (Lim et al., 2019). The feasibility of implementing MI in the clinical setting also warrants attention to the patients' perceived social support and quality of family relationships, as the interventions may differ depending on the level of involvement of significant others in the process of change.

## Conclusion

This scoping review leads to the conclusion that motivational strategies outperform traditional advice-giving in increasing adherence to CPAP use in patients with OSAS. However, a proper evaluation of the individuals' motivation to change and the provision of the corresponding motivational strategy in a clinical setting deserve further attention. Future trials providing more detailed information on the mechanisms of behavioral changes would help optimizing the effectiveness of motivational interventions in adults with OSAS. The cost-effectiveness of motivational intervention—alone or in combination with other interventions—might be a worthwhile endeavor to pursue.

## Author Contributions

GR and GP designed the study, conducted extensive literature searches, analyzed the data, and wrote the first draft of the paper. GM, IB, IT, EP, and CL revised the manuscript. GR, GP, GC, SG, FS, and PF reviewed methodological as well clinical issues and further edited the manuscript. All authors approved the final version of the manuscript.

## Conflict of Interest

The authors declare that the research was conducted in the absence of any commercial or financial relationships that could be construed as a potential conflict of interest.

## Publisher's Note

All claims expressed in this article are solely those of the authors and do not necessarily represent those of their affiliated organizations, or those of the publisher, the editors and the reviewers. Any product that may be evaluated in this article, or claim that may be made by its manufacturer, is not guaranteed or endorsed by the publisher.
