# Corrigendum: Improving CPAP adherence in adults with obstructive sleep apnea syndrome: A scoping review of motivational interventions

**DOI:** 10.3389/fpsyg.2023.1152441

**Published:** 2023-02-15

**Authors:** Giada Rapelli, Giada Pietrabissa, Gian Mauro Manzoni, Ilaria Bastoni, Federica Scarpina, Ilaria Tovaglieri, Elisa Perger, Sergio Garbarino, Paolo Fanari, Carolina Lombardi, Gianluca Castelnuovo

**Affiliations:** ^1^Department of Psychology, Catholic University of the Sacred Heart, Milan, Italy; ^2^Psychology Research Laboratory, Istituto Auxologico Italiano IRCCS, Milan, Italy; ^3^Faculty of Psychology, eCampus University, Novedrate, Italy; ^4^U. O. di Neurologia e Neuroriabilitazione, Istituto Auxologico Italiano IRCCS, Verbania, Italy; ^5^“Rita Levi Montalcini” Department of Neuroscience, University of Turin, Turin, Italy; ^6^Pulmonary Rehabilitation Department, Istituto Auxologico Italiano IRCCS, Verbania, Italy; ^7^Department of Cardiovascular, Neural and Metabolic Sciences, Sleep Disorders Center, Instituto Auxologico Italiano IRCCS, Milan, Italy; ^8^Department of Neuroscience, Rehabilitation, Ophthalmology, Genetics and Maternal-Infantile Sciences, University of Genoa, Genoa, Italy; ^9^Department of Medicine and Surgery, University of Milano-Bicocca, Milan, Italy

**Keywords:** sleep disorders, obstructive sleep apnea syndrome, continuous positive airway pressure, adherence, motivational intervention

In the published article, there was an error in the affiliations listed for author Giada Rapelli. Instead of affiliations 1 and 2, this author should only be affiliated to 1 - Department of Psychology, Catholic University of the Sacred Heart, Milan, Italy.

There was also an error in the affiliations listed for author Elisa Perger. Instead of affiliations 7 and 8, this author should only be affiliated to 7 - Department of Cardiovascular, Neural and Metabolic Sciences, Sleep Disorders Center, Instituto Auxologico Italiano IRCCS, Milan, Italy.

The remaining affiliation numbering has been amended as required in light of these changes.

The authors apologize for these errors and state that they do not change the scientific conclusions of the article in any way. The original article has been updated.

